# Official and private animal welfare inspectors’ perception of their own on-site inspections

**DOI:** 10.3389/fvets.2025.1575471

**Published:** 2025-04-25

**Authors:** Frida Lundmark Hedman, Ivana R. Ewerlöf, Jenny Frössling, Charlotte Berg

**Affiliations:** ^1^Department of Applied Animal Science and Welfare, Swedish University of Agricultural Sciences, Skara, Sweden; ^2^Department of Epidemiology, Surveillance and Risk Assessment, Swedish Veterinary Agency (SVA), Uppsala, Sweden

**Keywords:** assessment, control, experience, legislation, private standards, working environment

## Abstract

The presence of a trustworthy and effective animal welfare control system is important both for animal welfare and for public and consumer trust. The inspectors’ main task, regardless of whether they are official inspectors or private auditors, is to check for and enforce compliance with any relevant regulations. The aim of this study was to investigate how official animal welfare inspectors and private animal welfare auditors in Sweden perceive their inspection work and to explore any differences in the perception of being an inspector between these two groups. An electronic questionnaire was developed and received responses from 108 official inspectors and 22 private auditors (mainly inspecting the KRAV standard, Arlagården®, and the Trotter Health Standard). The results show that the official inspectors and private auditors usually enjoy their work, and they quite often have similar ambitions and views on what characterizes a good inspector. The respondents stated, for example, that it is important to have good dialog with the inspected animal keeper, that it is important to make uniform assessments (even if this can be challenging to achieve), and that animal keepers quite often show their appreciation after an inspection. However, there were also a number of differences in perception between the groups. For example, the official inspectors felt more exposed to unpleasant and threatening situations, while the private auditors were more likely to report the keeper being expected as acting nicely, professionally and relaxed during routine inspections. The official inspectors had a slightly more negative attitude toward the presence of private auditors than the other way around. Nevertheless, the respondents were in agreement that their collaboration and communication needed to be improved. One should bear in mind that the official inspectors also carry out inspections after complaints and more often make unannounced inspections. They not only inspect farms and horse premises, as the private auditors do, they also inspect different pet premises and have a secondary position of power as representatives of the government compared to the private auditors. These various circumstances may partly explain different views and perceptions between the official inspectors and the private auditors.

## 1 Introduction

All member states within the European Union (EU) are obliged to implement a system of official control to enforce the EU animal welfare legislation related to the keeping and management of farm animals, the transportation of live vertebrates and slaughter. The Commission’s Directorate-General for Health and Food Safety will, via the department for Health and Food Audits and Analysis (HFAA), ensure that this legislation is properly carried out (1). The Directorate-General publishes all audit reports on their webpage, including country profiles that describe how each EU member state has organized their official animal welfare control (2).

The EU directives on animal welfare have been implemented into the Swedish legislation, which consists of the Animal Welfare Act (2018:1192) issued by parliament, the Animal Welfare ordinance (2019:66) issued by the government, and national regulations issued by the SBA. In the ordinance, the Swedish government has regulated that the implemented system of official animal welfare control should be used also for other domestic animal related activities than farming and animal transport and slaughter. The Central Competent Authority in Sweden concerning animal welfare legislation and control is the Swedish Board of Agriculture (SBA) (2). The SBA issues national regulations concerning housing, management, transport and the slaughter of different types of animals, such as farm animals, companion animals, horses, zoo animals and animals used for research purposes. The on-site official animal welfare inspections are carried out by the regional County Administrative Boards (CABs), except at slaughterhouses, where the responsibility is shared between the CABs and the National Food Agency (2, 3). Sweden is divided into 21 counties, meaning that there are 21 CABs carrying out such inspections. The SBA provides guidelines and checklists for the CABs to achieve uniform implementation of legislation in different parts of Sweden. In 2021, the total annual national workforce amounted to approximately 230 official animal welfare inspectors at the CABs (4). The CABs carry out different types of inspection: risk-based standard inspections, random (*ad hoc*) standard inspections, acute inspections based on public or veterinary complaints, and cross-compliance inspections (5). The latter covers requirements originating from relevant EU legislation, and cross-compliance failure can lead to a reduction in EU subsidies for the animal owner (i.e., the farmers).

In addition to the legislation, there are private standards in the EU market (i.e., quality assurance schemes, labeling etc.) covering animal welfare and other areas (e.g., food safety, environmental protection) (6). In Sweden, there is, for example, the organic standard KRAV (farm animals), Arlagården® (dairy cows), Norrgården® (dairy cows), IP Sigill (dairy cows, beef cattle, lambs, pigs and poultry) and the Trotter Health Standard (trotting horses). Most of these are inspected by third party audit companies (i.e., independent external organizations), except for the Trotter Health standard, which is inspected by people who are employed directly by the Swedish Trotting Association (STA) (i.e., second party auditors). All private standards are based on legislation, but some of them have additional requirements related to animal welfare, such as the higher levels of IP Sigill and KRAV. The organic standard KRAV is the standard that differs most from the legislation, having a number of stricter animal welfare requirements.

Good animal welfare is considered important by citizens and consumers in Sweden, as well as in other countries (7). Having trustworthy and effective control is important both for animal welfare and for public and consumer trust (8, 9). Previous studies have indicated that a well-functioning control system is important for compliance with the legislation and other relevant standards (10). The inspectors’ main task, regardless of whether they are an official inspector or private auditor, is to check for and enforce compliance with the relevant regulations. Previous studies and investigations have shown that the level of compliance with animal welfare regulations can be improved, both in relation to EU legislation and in relation to Swedish animal welfare regulations (4, 8, 11, 12). Hence, it is important that the inspectors and auditors have satisfactory working conditions and sufficient resources to be able to carry out these inspections and enforce the regulation. A recently published paper from Finland revealed that Finnish veterinary inspectors working with official animal welfare control perceived their work as stressful, due to, for example, threatening situations and large amounts of overtime (13).

The aim of this study was to investigate how official animal welfare inspectors and private animal welfare auditors in Sweden perceive their inspection work in relation to their views on the controllability of the requirements, the handling of non-compliances, their own role, the inspected animal keepers’ roles, and the presence of both official and private animal welfare inspections. A second aim was to investigate plausible differences in the perception between official inspectors and private auditors, or differences in the perception based on age or gender.

## 2 Materials and methods

### 2.1 Questionnaire

An electronic questionnaire (see Supplementary material) asking official animal welfare inspectors and private auditors about their experiences and expectations related to animal welfare inspections was developed using the software program Netigate (version 8). The questionnaire was sent to all 254 official animal welfare inspectors in Sweden. Their email addresses were received from the CABs. The questionnaire was also sent to four private audit companies, where the managers forwarded it to the auditors working with animal welfare. In total 30 private auditors (four auditors from the STA, four auditors from HS Certifiering, ten auditors from Kiwa and twelve auditors from SMAK) received the questionnaire.

The questionnaire was open for four weeks in April/May 2021 and two reminders were sent out. The data received were analyzed anonymously. The study and the questionnaire were approved by the Swedish Ethical Review Authority (reference number: Dnr. 2019–6,370).

The questionnaire consisted of three parts: (1) information and background on the respondents and their professional role; (2) respondents’ views on the relevant animal welfare regulation, and their expectation and experience with performing inspections based on this regulation; and (3) respondents’ knowledge and views concerning other animal welfare regulations and inspections. The questionnaire consisted of 51 questions, mainly of the closed type but with some multiple-choice questions. The respondents were asked to choose from a list of options or state their opinion on a 5- or 10-point Likert Scale (1 = fully disagree and 5 = fully agree). There were also a few open-ended questions where the respondents could clarify their reply or express opinions without being given any pre-set options to choose between (see Supplementary material).

### 2.2 Data analysis

#### 2.2.1 Data preparation

Descriptive statistics and visualizations of the questionnaire responses were assessed to create an overview of the results. A decision was then made on how to prepare the questionnaire data for further statistical analysis.

#### 2.2.2 Statistical tests and analyses

The closed questions (which either gave a list of options or a Likert Scale) that were asked to both official inspectors and private auditors were analyzed further statistically. Responses given on a Likert scale were categorized into three categories (either ‘1–2’, ‘3’, and ‘4–5’, or ‘1–3’, ‘4–7’, and ‘8–10’). Responses given to questions with more than three possible options were also categorized into fewer (two or three) options (e.g., the age of the respondent was categorized into ‘0–40’ or ‘>40’). Pearson’s chi-squared or Fisher’s exact test (depending on response distribution) was then performed on 2×2 or 2×3 tables, comparing the responses between the two groups (official inspectors and private auditors). Furthermore, differences between the responses from female and male inspectors (excluding responses of “Other / Do not want to tell”) as well as between the two different age groups were also assessed separately, using one of the statistical tests on the 2×2 or 2×3 tables. Responses from the groups compared were considered as being significantly different with *p*-values below 0.05 from the test.

The open-ended questions were analyzed using qualitative thematic analysis, meaning that the free text answers were coded and categorized into different groups to summarize patterns and common views in the answers (14).

## 3 Results

### 3.1 Demographics and background information about respondents

Of the official inspectors who received a link to the questionnaire, 108 responded (a response rate of 43%). Of these, 98 submitted complete answers (i.e., answered all intended questions). The corresponding numbers for the private auditors were 22 respondents (a response rate of 73%), of which 21 submitted complete answers. The questionnaires of the respondents that did not complete all questions were still used in analyses where answers were provided. Of the private auditors, 77% (17/22) inspected the KRAV standard, 18% (4/22) the Trotter Health Standard, and 9% (2/22) Arlagården®. Almost half of the private auditors also inspected at least one other standard, such as IP Sigill or the European organic regulations. Official inspectors and private auditors from all 21 counties in Sweden participated. While each of the official inspectors worked in one county (i.e., region) only, it was common for the private auditors to cover several counties.

There were more female inspectors than male inspectors among the respondents (Table 1). The official inspectors were slightly younger on average than the private auditors, but the work experience in years of service was quite comparable (Table 1).

**Table 1:** Descriptive information on respondents.

		Official inspectors % (*n*)	Private auditors % (*n*)
Gender	Female	84 (91)	73 (16)
	Male	14 (15)	27 (6)
	Other / Do not want to tell	2 (2)	-
Age (years)	≤ 30	16 (17)	5 (1)
	31–40	43 (46)	9 (2)
	41–50	17 (18)	32 (7)
	51–60	18 (19)	27 (6)
	61–70	7 (8)	23 (5)
	˃ 70	-	5 (1)
Work experience	<5	29 (30)	32 (7)
(years)	5–9	24 (25)	18 (4)
	10–20	38 (39)	37 (8)
	21–39	10 (10)	14 (3)
Total (*n*)		100 (108)	100 (22)

The private auditors answered that they mainly inspected premises with beef cattle (82%, 18/22), sheep/goats (50%, 11/22), dairy cows (37%, 8/22), pigs (23%, 5/22) and horses (18%, 4/22), while the official inspectors mainly inspected premises with dogs (73%, 76/104), cats (72%, 75/104), beef cattle (66%, 68/104), horses (62%, 65/104), sheep/goats (50%, 52/104) and dairy cows (48%, 50/104). The respondents were allowed to select multiple answers, as most of them inspect different types of animal activities.

Most of the official inspectors (78%, 82/104) and private auditors (91%, 20/22) enjoyed being an inspector, i.e., stated that they were happy about their work (no significant difference between the two groups, *p* = 0.061).

### 3.2 The respondents’ basic and further education

Most official inspectors (98%, 105/108) and private auditors (82%, 18/22) had undergone education at a university level. The most common educational fields for the official inspectors were Animal Science (31%, 34/108), Ethology and Animal Welfare (26%, 28/108), and Environmental Health (20%, 22/108). The private auditors were mainly animal scientists (32%, 7/22) or had completed degrees in Agricultural and Rural Management (27%, 6/22). Most official inspectors (75%, 80/106) and private auditors (77%, 17/22) felt that they had the possibility of taking part in continuing education (i.e., the employer supports this). The main type of further training that the official inspectors had taken over the last five years had to do with threats and violence, communication skills, and assessments based on the legislation to increase inter-observer reliability. The courses that the private auditors had participated in during the same period focused on assessments based on private standards to increase inter-observer reliability, routines concerning inspection tasks, and general animal welfare. Of the respondents, 33% (34/104) of the official inspectors and 50% (11/22) of the private auditors stated that there was continuing education available to the extent needed. However, some of the private auditors explained that the main problem was not the range of courses available but rather the limited time to participate.

### 3.3 The respondents’ views on regulations and their controllability

Almost half of the private auditors (48%, 10/21) stated that they could influence the development and progression of a regulation, while 27% (27/101) of the official inspectors felt that they could influence the development of legislation. The proportion of private auditors who felt that there were requirements in the legislation that did not necessarily benefit animal welfare was 38% (8/21). Similarly, half (49%, 49/101) of the official inspectors were of the opinion that there were requirements in the legislation that did not necessarily benefit animal welfare in its current form, and also requirements that would certainly benefit animal welfare but that were currently not included in the legislation. The comments and examples given varied between the official inspectors but can be summarized into three categories: (1) Insufficient requirements based on the needs of the animals (e.g., horses and cattle being allowed to be kept tied, social animals allowed to be kept alone, too early weaning ages of piglets, and too restrictive pen space); (2) Lacking or not applicable requirements (e.g., there are no specific requirements for some species (e.g., Japanese quails), and for several production animals, the fact that the regulations are designed to fit large-scale production systems makes the regulations less suitable for small hobby farms); and (3) Illogical requirements that are hard to explain and motivate animal keepers to follow (e.g., requirements that originate from an EU directive and are perceived to be less relevant under Swedish farming conditions).

A majority (64%, 66/103) of the official inspectors stated that there were requirements in the legislation that are complicated and difficult to interpret; only 10% (10/103) felt the opposite. Of those stating that there were complicated requirements, most of them gave examples from the national regulations on horses, cats and dogs. Several of the official inspectors stated that the use of subjective words in the legislation leaves room for different interpretations. The inspectors wondered for example how “*enough*,” “*temporarily*,” “*a short while*” or “*in normal cases*” should be interpreted in different situations. One inspector wrote, “*It is difficult when the regulations are vague,* e.g.*, several measurement requirements have been removed from the horse regulation* [and replaced with more subjective and goal-oriented requirements – authors’ explanation]*. The horse owners want to comply with the requirements, but what are they exactly? It is difficult!*” Official inspectors also mentioned the difficulty in interpreting species-specific requirements in relation to the more general requirements stated in the Swedish Animal Welfare Act (SFS 2018:1192). They mentioned, for example, the requirement in the Act where all animals shall be kept in a way that enables them to perform relevant natural behaviors, while the inspectors must also assess more detailed species-specific requirements that may actually restrict some natural behaviors. For example, they are expected to accept tied cows (as this housing system is still legal), accept that ‘enough straw’ for pigs is a quite small amount, and that it still can be acceptable for horses to be kept alone (i.e., without the company of another horse). Significantly fewer (*p* = 0.009) private auditors (41%, 9/22) stated that the regulations they enforce were complicated and difficult to interpret. The most commonly mentioned difficulties had to do with the assessment of farm animals’ body condition and cleanliness. Both the official inspectors (67%, 69/103) and the private auditors (55%, 12/22) indicated that animal-based requirements were more difficult to assess than resource-based requirements. The inspectors/auditors who were above 40 years of age perceived the regulations as being easier to interpret than those who were younger (*p* = 0.001).

### 3.4 The respondents’ views on assessments and handling of non-compliances

The official inspectors and private auditors found it to be of great importance that they and their colleagues make uniform assessments, and a majority of inspectors stated that they should not have much room for interpretation, although the private auditors wanted more room for interpretation than the official inspectors (Table 2). However, only slightly less than half of the official inspectors and private auditors believed that they usually made completely uniform assessments with their colleagues (i.e., inter-observer agreement). A larger proportion felt that it is easy for them to make uniform assessments between different animal premises (i.e., intra-observer agreement). Some of the respondents agreed with the statements that they might accept minor non-compliances if animal husbandry and welfare was generally good, and a larger proportion of the private auditors stated that non-compliances affecting several animals in a herd was more serious than if it only concerned single animals (Table 2).

**Table 2:** The proportion of official inspectors and private auditors who agree or disagree with statements related to uniform assessments and handling of non-compliances.

Statement	Official inspectors (*n* = 100–103)		Private auditors (*n* = 21–22)		
	Agree	Disagree	Agree	Disagree	*p*-value
It is important that we as inspectors make uniform assessments	98%	0%	100%	0%	*p* = 1
My colleagues and I usually make uniform assessments	47%	17%	45%	18%	*p* = 1
It is easy for me to make uniform assessments between different animal premises	69%	2%	64%	5%	*p* = 0.536
Inspectors should have considerable room for interpretation so that good solutions are found in individual cases	8%	49%	24%	57%	*p* = 0.034*
Inspectors should not have much room for interpretation since it leads to too much ‘special treatment’ of different animal keepers	60%	8%	58%	24%	*p* = 0.086
I usually take the opportunity to accept minor non-compliances related to measurement requirements if the animal husbandry on a premises is generally good ^a^	51%	15%	-	-	-
I sometimes accept minor non-compliances if the animal husbandry on a premises is generally good ^b^	20%	49%	41%	36%	*p* = 0.095
It is acceptable to only give a verbal statement for minor non-compliance without noting it in the checklist or in the inspection report	13%	66%	19%	71%	*p* = 0.453
Non-compliance is non-compliance regardless of severity, so I handle them equally	36%	42%	48%	24%	*p* = 0.309
Non-compliance affecting several animals in a herd is more serious than if it concerns a single animal	36%	29%	77%	15%	*p* = 0.003*
We have predetermined time spans for when certain types of non-compliance must be rectified	11%	72%	76%	5%	*p* < 0.001*
Most animal keepers understand what is written in the inspection report after an inspection	74%	2%	76%	5%	*p* = 0.582
We always follow up that the non-compliance has been remedied	38%	43%	72%	15%	*p* = 0.014*

The respondents graded their statements from 1 = totally disagree to 5 = fully agree, where 1–2 were categorized as “disagree” and 4–5 as “agree” in the table; grade 3 (neutral) has been omitted. The table also shows if there was a significant difference (*p*-value < 0.05, *) or not between the two groups for each statement.

^aThe legislation sometimes allows official inspectors to, under certain circumstances, accept minor deviations from the measurement requirements.^

^bIn the questionnaire to the official inspectors it was clarified that this statement has do to do with other non-compliance than those related to measurements.^

The respondents were also asked to grade the amount to which different factors influenced their assessments, decisions and handling of cases in relation to an animal welfare inspection (Table 3). Not surprisingly, the vast majority of official inspectors stated that the actual requirements written in the legislation had a great impact on the assessments, decisions and handling of cases. A majority of the private auditors stated the same, but to a lower extent than the official inspectors (*p* < 0.001). The private auditors graded guidelines as more important for their decision making than what is stated in the actual regulations (Table 3). Both official inspectors and private auditors seemed to use the available inspection guidelines to a great extent, both the guidelines from the central authority/owner of the regulation and guidelines developed by several CABs or control bodies together, and guidelines developed by their own CAB or control body (Table 3). Discussions with colleagues were also reported to be of high importance for assessment and decision making for both the official inspectors and private auditors (Table 3). The attitude of the animal keeper during an inspection did also matter to some extent (e.g., the animal keeper’s expression of understanding and willingness to correct non-compliances found), especially for the official inspectors (*p* = 0.014) (Table 3).

**Table 3:** The proportion of official inspectors and private auditors answering on the extent to which different factors affected their assessments, decisions and handling of cases in relation to an animal welfare inspection.

Factor	Official inspectors (*n* = 100)		Private auditors (*n* = 21)		
	None or a very small impact	Large impact	None or a very small impact	Large impact	*p*-value
The actual requirements	1%	96%	15%	58%	p < 0.001*
The regulation’s aim and intentions	3%	78%	15%	77%	*p* = 0.063
Written guidelines from the central authority/owner of the regulation	7%	76%	10%	81%	*p* = 0.625
Verbal guidance from the central authority/owner of the regulation	45%	31%	15%	52%	*p* = 0.033*
Guidelines developed by several CABs or control bodies together	9%	63%	0%	71%	*p* = 0.148
Guidelines developed by my own CAB or control body	8%	78%	0%	86%	*p* = 0.335
Discussions with my colleagues	0%	94%	0%	81%	*p* = 0.07
The animal keeper’s attitude and ability to engage in dialog	36%	29%	39%	24%	*p* = 0.89
The animal keeper’s competence regarding animal welfare	27%	34%	48%	19%	*p* = 0.15
The animal keeper’s understanding and willingness to rectify non-compliance	12%	63%	38%	38%	*p* = 0.014*
Experience from previous inspections with this animal keeper	12%	55%	19%	67%	*p* = 0.191
The general level of animal husbandry at the time of inspection	11%	60%	14%	62%	*p* = 0.777
The inspector’s ‘shape of the day’	82%	2%	77%	0%	*p* = 0.678

The respondents graded their statements from 1 = no impact to 5 = very large impact, where 1–2 were categorized as “none or a very small impact” and 4–5 as “a large impact,” shown in the table. The table also shows any significant differences (*p*-value < 0.05, *) between the two groups for each statement.

More than half of the official inspectors (56%, 56/100), and a majority of the private auditors (81%, 17/21) did not feel that discussions regarding their assessments and interpretations occurred often when carrying out planned standard inspections. Ten percent of the official inspectors (10/100) and the private auditors (2/21) had experience of often ending up in such discussions. Furthermore, younger respondents (i.e., those under 40 years of age) seemed to end up in discussions regarding their assessments more often than their older colleagues (*p* = 0.007).

### 3.5 The respondents’ views on inspection purpose, routines and their own role

A majority of the respondents (official 89%, 87/98; private 86%, 18/21) stated that it was reasonable that an animal premises is inspected by either CAB or a private audit company at least every third year regarding animal welfare. However, the private auditors stated to a higher extent than the official inspectors did that an animal welfare inspection should be carried out at least once a year (*p* = 0.03). Both the official inspectors (95%, 95/100) and private auditors (95%, 20/21) reported that their main task during an inspection was to check for compliance with the regulation. A majority of the respondents (official 87%, 87/100; private 81%, 17/21) agreed with the statement that they are allowed to provide information during an inspection so that the animal keeper understands the intention and purpose of the regulation. Quite a high percentage of the respondents (official 64%, 64/100; private 48%, 10/21) also stated that one purpose of the inspections was to help the animal keepers to comply with the regulation. The official inspectors did agree to a greater extent (*p* = 0.008) that they, as part of their role as inspector, are allowed to give animal keepers advice on the measures that need to be taken in order to comply with the regulation. Almost half of the official inspectors (46%, 46/100) agreed with this statement, while 20% (20/100) disagreed. The corresponding number for private auditors was 28% (6/21, agreed) and 52% (11/21, disagreed). A smaller proportion of both official inspectors (24%, 24/100) and private auditors (24%, 5/21) agreed with the statement that they are allowed to give animal keepers advice so that animal husbandry can be improved above the level of regulation.

**Figure 1:**
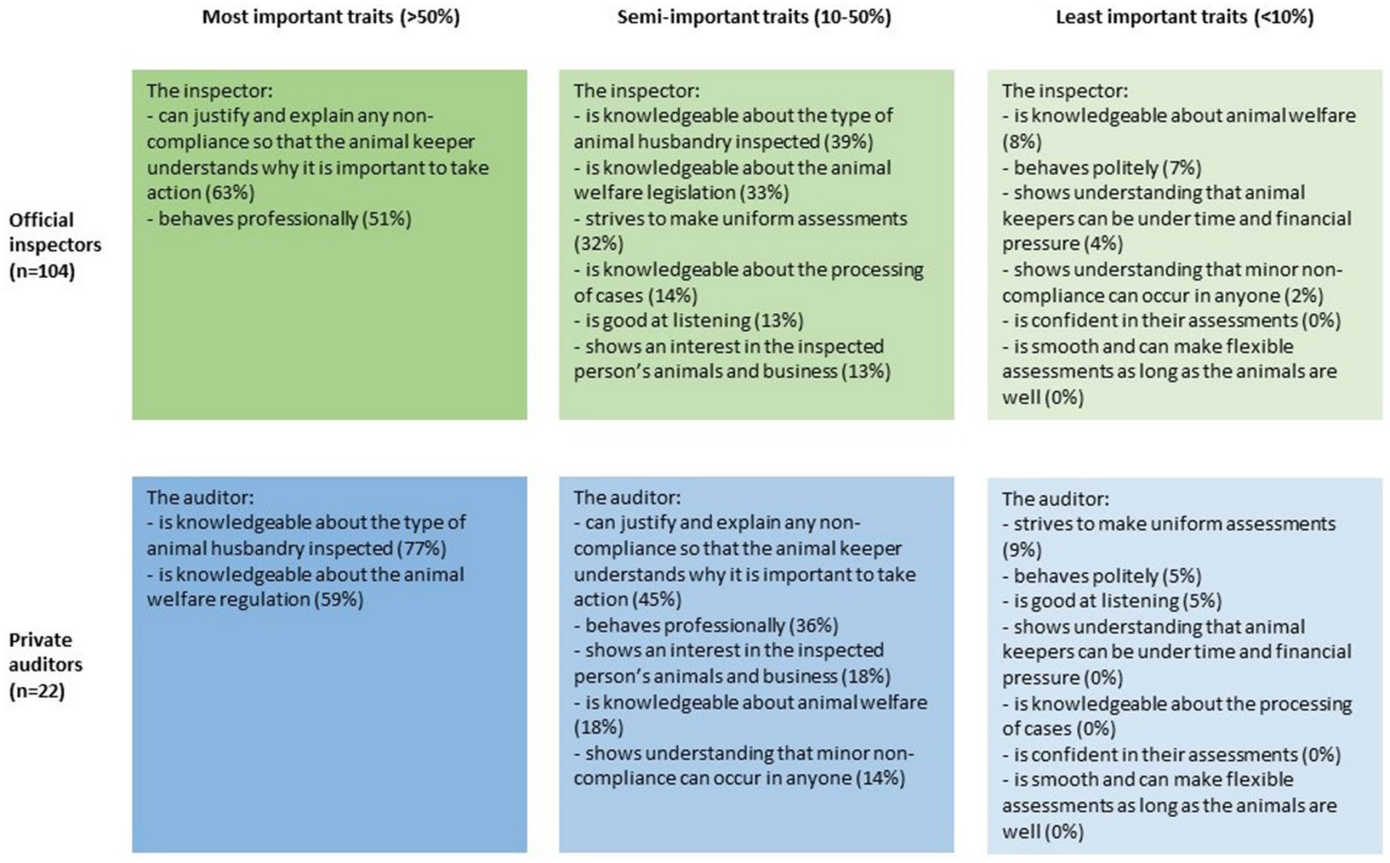
The official inspectors’ and private auditors’ view on what they thought characterized a good animal welfare inspector/auditor. Their task was to state which three traits they believe were most important.

The official inspectors and private auditors had the same top four traits (but in a different order) that they thought characterized a good inspector (Figure 1). The traits that both the official inspectors and the private auditors ranked lowest were that the inspector is confident in their assessments, and that the inspector is smooth and can make flexible assessments as long as the animals are doing well.

### 3.6 The respondents’ views on treatment and communication

The vast majority of the official inspectors (93%, 93/100) and all private auditors (100%, 21/21) stated that good dialog with animal keepers was a prerequisite for good inspection work. Furthermore, most respondents stated that they were satisfied with an inspection when the dialog with the animal keeper has been good (official 98%, 98/100; private 95%, 20/21), and when the animal keeper seems to have understood what has been communicated during the inspection (official 99%, 99/100; private 100%, 21/21). However, the official inspectors (57%, 57/100) felt to a greater degree than the private auditors (29%, 6/21) that conflicts with animal keepers were a barrier to improving animal welfare (*p* = 0.003). This was also significant for age, with younger respondents (i.e., younger than 40 years) reporting that conflicts were a barrier to a greater degree than those above 40 years of age (*p* = 0.009). Both the official inspectors (95%, 95/100) and private auditors (95%, 20/21) responded that it was important to create a safe and open atmosphere during an inspection. However, the private auditors (91%, 19/21) were more likely to feel that the inspected animal keepers were calm and relaxed during a planned standard inspection than the official inspectors (51%, 51/100) did (*p* = 0.003).

Approximately half of the respondents agreed that unannounced inspections promoted animal welfare more effectively than pre-notified inspections (official 47%, 47/100; private 48%, 10/21). However, the respondents agreed that pre-notified inspections contributed to a more pleasant atmosphere and better dialog with the animal keeper (official 76%, 76/100; private 76%, 16/21).

Many of the respondents (official 62%, 62/100; private 67%, 14/21) stated that animal keepers often showed appreciation after an inspection.

### 3.7 The respondents’ perceptions of the inspected persons and their animal premises

All private auditors agreed (100%, 21/21) that most animal keepers would act nicely and professionally during a planned standard inspection, in comparison to 74% (74/100) of the official inspectors (*p* = 0.014). All private auditors agreed (100%, 21/21) with the statement that most of the inspected people receiving a planned standard inspection in general kept and managed their animals well. There was a lower proportion of official inspectors (66%, 66/100) who agreed with this statement (*p* = 0.002). Furthermore, the majority of the respondents (official 73%, 73/100; private 100%, 21/21) stated that most animal holdings did not show severe non-compliance, even if the official inspectors disagreed more often with this statement (*p* = 0.019).

The majority of the respondents stated that most animal keepers had the intention of being law abiding and complying with the regulations (official 70%, 70/100; private 86%, 18/21), while 4% (4/100) of the official inspectors disagreed with this statement. However, only about a third of respondents (official 30%, 30/100; private 38%, 8/21) stated that most animal keepers had good knowledge of the regulations. In addition, the official inspectors agreed less (51%, 51/100) with the statement that most animal keepers have good knowledge of the animals’ needs and welfare, compared to 81% (17/21) of the private auditors (*p* = 0.038). The main reasons for farmers and horse keepers lack of compliance with a regulation was, according to both the official inspectors and private auditors, a lack of knowledge concerning the regulation and a lack of practical and financial ability to comply with the regulation. According to the official inspectors, these were also common reasons for pet keepers’ non-compliance. In addition, the official inspectors perceived that it was more common for pet keepers to show non-compliance due to a lack of knowledge concerning the animals’ needs as well as personal or psychological problems, compared to people keeping farm animals and horses.

### 3.8 Respondents’ experiences of difficult situations in relation to inspections

The majority (69%, 69/100) of the official inspectors stated that it was more demanding to handle animal welfare cases that are based on complaints than to carry out planned standard inspections. It was more common for an official inspector to have felt afraid during an animal welfare inspection or to have been exposed to threats or violence than for a private auditor (Table 4). It was also more common that younger inspectors and auditors (<40 years of age) reported having been afraid during an inspection than their older colleagues (*p* < 0.001). Almost half of the official inspectors (46%, 31/67) stated that their experience of threats or violence had affected how they later processed and assessed a case. The majority (80%, 55/69) of the official inspectors that reported having been exposed to threats or violence also stated that their employer (i.e., the CAB) had reported some of the incidents to the police or a prosecutor (i.e., the judicial system). Of the private auditors that had been exposed to threats, none of them (0%, 0/4) stated that the incident had been reported to the judicial system.

**Table 4:** The proportion of official inspectors and private auditors answering whether they had experienced difficult situations in relation to an inspection.

	Official inspectors (*n* = 100)			Private auditors (*n* = 21)			
	Never	Sometimes	Often	Never	Sometimes	Often	*p*-value
I have been afraid during an inspection	12%	83%	5%	71%	29%	0%	*p* < 0.001*
I have been exposed to threats	15%	82%	3%	71%	29%	0%	*p* < 0.001*
I have been exposed to physical violence	79%	21%	0%	100%	0%	0%	*p* = 0.023*
My competence has been questioned during an inspection	3%	77%	20%	33%	67%	0%	*p* < 0.001*
I have received disparaging comments regarding my age or gender	25%	65%	10%	81%	19%	0%	*p* < 0.001*
I have seen severe animal suffering	1%	63%	36%	19%	81%	0%	*p* < 0.001*
I have seen people (i.e., animal keepers) in very difficult situations	0%	58%	42%	5%	95%	0%	*p* < 0.001*

The respondents graded their statements on a five-grade scale from “never” to “very often,” which is aggregated and categorized as follows in the table: “never” (never), “sometimes” (sometimes + quite often), and “often” (often + very often). The table also shows whether there was a significant difference (*p*-value < 0.05, *) or not between the two groups of respondents for each statement.

The official inspectors reported having received disparaging comments regarding their age and gender more often than the private auditors did (Table 4). In addition, it was more common that such comments were given to female inspectors or auditors (*p* < 0.001) compared to male, and those younger than 40 years of age (*p* < 0.001) compared to older respondents, which could explain the difference between official inspectors and private auditors. The official inspectors’ competence had also been questioned by the animal owners more often than the private auditors’, but the majority of both types of inspectors reported that this had happened to them (Table 4). The experience of having had their competence questioned was also more common for the younger respondents (<40 years of age) than for those above 40 years of age (*p* < 0.001).

Most respondents (official 82%, 82/100; private 62%, 13/21) reported that their employer had routines and protocols in place for how to handle threats and violence. A higher percentage of official inspectors stated that they had such routines and protocols than private auditors (*p* = 0.011). The official inspectors also reported to a higher degree that their employer (i.e., the CABs) had routines and protocols on how to handle difficult situations during an inspection, such as encountering animal or human suffering, or being contested as an inspector (*p* = 0.024). Half of the official inspectors (50%, 50/100) and a third of the private auditors (33%, 7/21) agreed with having such routines and protocols in place.

Neither the official inspectors, nor the private auditors agreed to any high extent that what media reports in relation to animal welfare inspections and audits were in line with reality, as 81% (17/21) of private auditors disagreed with the statement and 50% (50/100) of the official inspectors disagreed, while 44% (44/100) were neutral. About a quarter of official inspectors (26%, 26/100) reported having been negatively mentioned by name on social media in connection with an animal welfare inspection. The corresponding number for the private auditors was 5% (1/21), which was significantly lower (*p* = 0.023). It was also more common that the older respondents (>40 years of age) reported having been negatively mentioned on social media (*p* = 0.018). However, some official inspectors (7%, 7/100) and some private auditors (20%, 4/21) reported having been mentioned by name in a positive way after an inspection on social media.

### 3.9 Respondents’ views on the presence of both official and private animal welfare inspections

Half of the private auditors (52%, 11/21) and 41% (41/99) of the official inspectors stated that it was necessary to have both official and private animal welfare inspections. However, the official inspectors were less likely to feel that the presence of private audits was beneficial for animal welfare (*p* = 0.016), as 43% (43/99) of the official inspectors did not agree to this statement while 77% (16/21) of the private auditors did. The official inspectors’ main concern, according to their free-text answers, regarding the presence of private audits was the perception that private auditors too often ignored obvious animal welfare problems, leading to different assessment outcomes between an official inspection and a private audit, which could be difficult to understand for the animal owner. This quote from one CAB inspector reflects what several official inspectors stated: “*Unfortunately, it is very common that obvious or major animal welfare deficiencies are noticed by us on farms that have recently been inspected by a private audit company, which means that I, as an official inspector, have little confidence in the private audits*.” Some official inspectors also questioned the impartiality of private audits as these are based on financial interests and are run by the industry themselves, albeit with second- or third-party audit companies.

The official inspectors were more likely to experience animal keepers having difficulties differentiating between official inspections and private audits (*p* = 0.017). Only 11% (11/99) of the official inspectors felt that it was easy for the animal keepers to differentiate between official and private inspections, while 64% perceived it as being difficult. However, the private auditors had also seen difficulties as almost half of them (48%, 10/21) did not feel it to be easy for the animal keepers to keep track of different inspections, while 39% felt that it was easy.

The official inspectors seemed to be less aware of similarities and differences between the legislation and private regulations when it came to the requirements and their assessment (*p* < 0.001). Only 7% (7/99) of the official inspectors stated that they were well aware of such similarities and differences. The corresponding number for the private auditors was 66% (14/21). The official inspectors were much more likely to disagree that official inspectors and private auditors generally made the same assessment of similar requirements, i.e., that the outcome of an assessment would often be the same (*p* < 0.001). Only 3% (3/99) of the official inspectors agreed with this statement, while half (52%, 11/21) of the private auditors agreed. The majority of both the official inspectors (71%, 71/99) and private auditors (62%, 13/21) disagreed with the statement that private auditors made stricter animal welfare assessments than the official inspectors, while only 3% (3/99) of the official inspectors and 15% (3/21) of the private auditors agreed with the statement. A larger proportion (*p* < 0.001) of the private auditors (43%, 9/21) stated that they usually informed the animal keeper that an official inspector might give an assessment that differed with theirs, while 16% (16/99) of the official inspectors agreed with the statement that they usually informed animal owners that private auditors may make different assessments.

Both official inspectors and private auditors stated that communication and collaboration between them was scarce. Most of them (official 95%, 93/98; private 100%, 21/21) stated that they did not synchronize any inspections to avoid inspections being close in time at the same animal premises. Most (official 93%, 91/98; private 100%, 21/21) did not carry out joint inspection/s to decrease the number of inspections at the same premises. The private auditors stated more often that they had a routine of contacting the CAB if substantial animal welfare problems were detected during an inspection, than the other way around (*p* = 0.003). Of the private auditors, 38% (8/21) stated that they had such routines, while only 3% (3/98) of the official inspectors stated that the CAB had a routine of informing any relevant private audit company if substantial animal welfare problems were detected during an official inspection. However, only 1% (1/98) of the official inspectors agreed with the statement that the private audit companies usually contacted the CAB if non-compliance was detected during a private inspection. Of the private auditors, 5% (1/21) perceived that CAB usually contacted them if non-compliance was identified during an official inspection. The majority of both official inspectors (63%, 61/98) and private auditors (86%, 18/21) stated that they would like to see better collaboration between the CABs and the private audit companies.

## 4 Discussion

The aim of this study was to investigate how official inspectors and private auditors in Sweden perceived their animal welfare inspection work and whether there were any differences in the perception between these two groups. Previously, two equivalent studies have been carried out on how animal keepers in Sweden (i.e., dairy farmers and trotting horse trainers) perceive being inspected by official inspectors and private auditors (15, 16). Therefore, some comparisons will be made with these studies in the discussion below.

### 4.1 Respondents’ working environment

The official inspectors and the private auditors generally liked their work, and most of them stated that they had often been shown appreciation by animal keepers after an animal welfare inspection. The respondents also pointed out the importance of good dialog with the animal keepers and their own intention to create a calm atmosphere during the inspections. According to the respondents, it seems like the private auditors managed to create a calm atmosphere more often than the official inspectors. However, it is not known if this outcome is mainly related to the efforts of the inspectors/auditors, or perhaps to differences in attitudes and willingness to communicate on the side of the animal keepers. This result is in line with the previous study, where dairy farmers stated that they were especially worried before an official inspection and less worried before a KRAV audit (15). Having good and constructive dialog with the animal keepers during animal welfare inspections has also been mentioned as being important by, for example, Norwegian official inspectors (17), French official inspectors (18), and Danish official inspectors (19). A constructive dialog between the inspector and the animal keeper has also been reported to be important, but sometimes challenging, by animal keepers (15, 16, 20, 21).

In addition, the results imply that younger women carrying out animal welfare inspections receive a greater number of disparaging comments from animal keepers, than their older male colleagues. It was also more common that younger inspectors/auditors had felt afraid during an inspection and had experienced having their competence questioned. In this study, the official inspectors were, on average, somewhat younger compared to the responding private auditors, and a slightly higher proportion of the official inspectors were female compared to the private auditors. These differences, both in age and gender, could potentially explain some of the observed differences in perception between the official inspectors and private auditors. Previous studies have shown that it can be more challenging to be a female inspector and that this is connected to the power relations in society (22, 23). A previous study showed that Swedish dairy farmers’ perceptions of animal welfare inspections were more negative if the official inspector was a woman younger than themselves (15).

Being employed by the government inherently implies a certain position of power. This can be one of the reasons behind some of the different experiences related to the working environment noted between the official inspectors and private auditors. It has previously been shown that the Swedish official animal welfare inspectors are exposed to a considerable number of threats and violence (24, 25). As an example, in 2023, 18 out of 21 CABs received a threatening letter signed ‘The animal owners in Sweden’ addressed to the animal welfare units at the CABs (26, 27). The letters contained an unidentified white powder, which was initially suspected to be dangerous, although a chemical analysis later revealed that it was harmless. In a more recent survey conducted by the SBA, 82% of the official animal welfare inspectors reported having been exposed to threats, 9% to physical violence, 26% to attempted physical violence, and 92% reported attempts to influence their decision and handling of a case (e.g., by the animal keepers threatening to harm themselves, contact the media, or the inspector’s superior) (28). Animal keepers threatening to hurt themselves, and even commit suicide, has also been reported in Ireland (29). Almost half of the Swedish official inspectors in this study stated that their experience of threats or violence had affected the subsequent processing of a case and the final assessment. However, we do not know in what way the process and assessment was affected, i.e., if it influenced the decision on whom (which individual employee) that was to continue processing the case, the way the case was documented, the time it took to process the case, the type of feedback given to the animal keeper or the actual outcome of the case. In a recently presented investigation ordered by the Swedish government, there are suggestions on how to handle threats, violence and harassments toward public employees (30). The overall picture of the investigation is that the vulnerability among public employees, particularly to threats and harassment, is extensive and appears to have increased. The investigation suggested several actions to be taken, such as more severe penalties for exposing a public employee to threats or violence and a new crime category of ‘insulting’ a public employee. In this study, the high risk of exposure to threats and violence as an official inspector was also reflected by the fact that this was the theme for most of the further education courses that the official inspectors had been taking, and the CABs had more routines and protocols for handling such situations than the private audit companies. It has also been shown in other countries [Canada (31), Poland (32), and Finland (13)] that it can be a risky business to enforce the animal welfare legislation. The official animal welfare inspectors in Finland suggested that they should be given the ability to work in pairs more often, as this would feel more secure (13). Swedish official inspectors do regularly, but not always, work in pairs for different reasons, such as increasing inter-observer reliability, training of new inspectors, or safety (15, 33).

There are also other possible reasons for the different experiences of unpleasant situations between official inspectors and private auditors. The official inspectors carry out many inspections of pets (companion animals), which the private auditors do not. It is quite common that animal welfare violations are related to pet animals. Last year, 78% of all animal welfare cases that led to a seizure of animals in Sweden involved dogs or cats (28). Animal welfare violations related to pets are also an increasing problem in other countries, e.g., Finland (34). The reasons behind pet inspections in Sweden are often complaints (from the public, a veterinary clinic, etc.), and the CABs have a routine of making such inspections unannounced to prevent the risk of the owner hiding the animal prior to a pre-announced inspection. Official inspectors have reported that threats and violence are most common at pet premises (28). However, this does not imply that no problems of this kind are found at other types of animal premises or with other inspected people.

Another circumstance that might affect the different perceptions between the respondents is that the CABs’ planned standard inspections are risk-based (according to EU Regulation 2017/625 of the European Parliament and of the Council on official controls and other official activities performed to ensure the application of food and feed law, rules on animal health and welfare, plant health and plant protection products), i.e., the inspections shall be carried out where the risks for non-compliances and poor animal welfare is greatest. Hence, it is not a random selection, and animal keepers who have previously had problems complying with animal welfare legislation tend to receive inspections more often. This may also be the reason why the official inspectors in this study perceived the animal keepers to have a poorer animal management level and greater lack of competence, as well as having seen more severe animal suffering compared to the private auditors.

Regardless of the reason, inspectors and auditors need to have a safe working environment so they can enforce animal welfare regulations without being afraid of unpleasant consequences. Having a poor working environment can negatively affect the employees’ motivation and performance, and hence, the quality of their work (35, 36). A poor working environment can also increase the risk of stress related health problems (37, 38). Job satisfaction is important to minimize employee turnover, a turnover which is costly both in terms of money and competence (39). Therefore, job satisfaction and the working environment of inspectors and auditors are important for themselves, for the quality of the inspections and audits they perform, and for the sake of the inspected animal keepers. Hence, the working conditions for inspectors and auditors will be of importance for animal welfare in a country.

### 4.2 Respondents’ views on their own role

According to Swedish dairy farmers and trotting horse trainers, their level of satisfaction with an animal welfare inspection is dependent on how they perceive the inspector’s competence, manner, and behavior (15, 16). The results in this study show that the inspectors and auditors are aware of these expectations as they emphasized the importance of their own actions and behavior. Worth noticing is that there is formally no common and mandatory education or training for animal welfare inspectors and auditors in Sweden. However, nowadays most animal welfare inspectors and auditors have a three-year university education covering biology, legislation and inspection methodology. For inspectors with other backgrounds, continuous professional development (CPD) courses are available. This differs from some other countries where, for example the official inspections are carried out only by licensed veterinarians. The EU Commission, via Better Training for Safer Food, BTSF, (https://better-training-for-safer-food.ec.europa.eu/training/) and the EU Reference Centers for animal welfare, EURCAW (https://food.ec.europa.eu/animals/animal-welfare/eu-reference-centres-animal-welfare_en) offers CPD mainly directed toward official animal welfare inspectors. There are also international private initiatives for third party auditors, for example, the Professional Animal Auditor Certification Organization (https://animalauditor.org/) that offers education and certification for auditors, but these are rarely, to our knowledge, used in the EU. The inspectors and auditors in this study largely agreed with each other regarding what traits made a good inspector, even if there were some minor differences. However, there seems to be some obvious differences in the perception of what constitutes a good animal welfare inspector between the official inspectors and private auditors on one hand, and the dairy farmers and trotting horse trainers on the other hand. The animal owners graded inspectors who could make flexible assessments higher (15, 16), a trait that the inspectors and auditors in this study stated to be one of the least important. The respondents in this study (especially the private auditors) did, however, agree with the dairy farmers and trotting horse trainers that it is important that the inspector is knowledgeable and familiar with the type of animal husbandry inspected. It is important that the similarities and differences between the different views of the inspectors’ role are discussed and illuminated in order to generate shared expectations.

The respondents in this study reported that their main task during an inspection is to check for compliance with a regulation, and that this task includes providing information to the animal keeper so they will understand the regulation. The official inspectors stated to a high degree that they could give animal keepers advice on what measures needed to be taken in order to comply with the legislation. This is in contrast with what Danish official inspectors have stated, that giving advice is not allowed as it can be used against the inspector in a later court case (19). This difference may partly be based on how the term ‘advice’ is defined by the respondents, as the Swedish inspectors are not expected to or allowed to formally advise animal owners, for example, on how to construct buildings, what brand of equipment to buy or what routines to implement. However, they can give information and guidance about the current requirements in the legislation and examples related to problem solving. Receiving good advice is important for the dairy farmers’ and trotting horse trainers’ positive perception of an inspection. However, neither the trotting horse trainers nor the dairy farmers reported having been given more advice from the official inspectors than any private auditor, but rather the opposite (15, 16). Hence, there is a risk that there are different expectations and definitions on what guidance in relation to compliance with regulation actually means.

### 4.3 Views on uniformity and the controllability of the regulations

The respondents in this study stated that making uniform assessments is important. However, fewer of them stated that they are able to achieve this.

Firstly, the respondents did report that there are requirements in the regulations that are difficult to interpret. However, the official inspectors seemed to have more trouble with interpreting the legislation. The official inspectors mentioned that the newer national regulations for dogs, cats and horses have been made more flexible and goal oriented, which has made interpretation and uniform assessments more difficult. It has been argued that the use of vague formulations in regulations increases the risk of variation in interpretations (33, 40–43). This has also been noticed by the EU Commission, which has concluded that it is important to have clearly formulated animal welfare requirements to avoid disparities and to better protect animal welfare (8). In Sweden, both the government and the farming industry have recently tried to influence the SBA to make more goal oriented and flexible regulations (44). At the same time, they want to see clear and unambiguous regulations. Hence, there are certainly mixed signals on how the regulations should be designed.

Secondly, the results show that both the official inspectors and private auditors sometimes do accept minor non-compliance if they assess the overall animal husbandry and welfare to be good. This is in agreement with Anneberg et al. (19), who reported Danish animal welfare inspectors stating that their decision to note a minor non-compliance or not varies, and that it is not possible to fully standardize inspections. In our study, we found disparate opinions among the respondents regarding how much room for interpretation the inspectors and auditors should have. However, the private auditors were more likely to feel that inspectors/auditors should have considerable room for interpretation so that good solutions can be found in individual cases. Holm (45) found that the Norwegian official animal welfare inspectors that perceived their role to be more indicative and consultative than police-like had a more positive attitude toward considerable rooms for interpretation. There are no easy answers to how much discretion (i.e., room for maneuver when it comes to following or deviating from rules and procedures to address client needs and circumstances) that should be allowed or that are preferred, but some level of discretion may be necessary when handling complex issues (46–48). However, research on street-level bureaucracy has suggested that when there is much room for interpretation, discretion factors, such as age, gender, working experience, personal norms, and education, may affect the assessment and outcome (33, 48, 49). This is important to bear in mind as the inspectors and auditors working in the field will be of different ages, genders, etc. and will have different educational backgrounds and work experience.

Thirdly, a reason behind the difficulties in achieving uniformity could be the presence of unclear guidelines, i.e., documents in addition to the basic regulation. Even if an inspection guideline aims to increase uniformity, this will be hard to reach if the guidelines themselves leave room for maneuver, which some of the guidelines do (50). A guideline or checklist can also be detailed and simultaneously imprecise, leading to subjective assessments (18). Another reason behind the difficulties in achieving uniformity is that the individual CABs and the private audit companies tend to develop their own inspection guidelines. Our results imply that this is common, and that these guidelines are used to a large extent, giving them a substantial impact on the assessment, handling and decisions related to an inspection. Veissier et al. (18) found that it was common for French official animal welfare inspectors to create and use their own criteria in addition to an official checklist. The main reasons for this seemed to be a perception or opinion that certain criteria were missing from the official checklists. Regardless of the reason for individual guidelines and criteria, there is a risk that different assessments of similar situations will be made between colleagues placed in different parts of the country, while still carrying out inspections based on the same regulation. Another risk is that precise guidelines can be perceived as containing additional requirements, although they do not constitute binding law. Hence, it is important that animal keepers are aware of such guidelines, for transparency and from a legal security perspective.

Finally, we have the presence of both official inspections and private audits, which leads us to the question of uniformity between the different actors (i.e., official and private). Most private standards in Sweden regarding animal welfare contain more or less the same or similar requirements as the animal welfare legislation, at least as a baseline. However, the inspection guidelines and the assessments will not necessarily be the same between these different regulations. Previous studies have illustrated the risk of different outcomes when using different guidelines and assessment protocols (11, 40, 50, 51). In this study, the majority of the official inspectors perceived that they and the private auditors do not note the same finding in similar situations when applying the same requirements. This was also the perception of some of the private auditors, but to a lower extent. Previous Swedish studies have also shown that dairy farmers especially have experienced such differences in outcome between inspections carried out by official inspectors and private auditors (15).

It might be expected that the respondents would perceive the private auditors as often making stricter animal welfare assessments than the official inspectors, since the legislation represents the minimum standard and the private standards usually, more or less, aim for a higher animal welfare level. However, this seems not to be the case, as the respondents were in agreement that private auditors do not make stricter assessments. In previous studies, we have, for example, seen that the outcome will differ depending on whether the assessment is carried out at the individual or group level, i.e., if every single animal matters or only an average figure is sought (11, 50, 52). In those studies, it was more common for the private auditors to measure at a group/herd level than the official inspectors. This may also be reflected by the results of this study, where the majority of private auditors stated that non-compliance affecting several animals in a herd was more serious than if they concerned individual animals only. Winckler (53) illustrated that group-based animal welfare assessments are often used within private assurance schemes, while pointing out the fact that animal welfare in general refers to an individual animal’s state and experience.

### 4.4 The presence of both official and private inspections

The private auditors reported having more knowledge related to the official animal welfare inspections and the official legislation, than the other way around. This can probably be explained by the private standards being based on the legislation. In addition to having less knowledge about private audits, the official inspectors also had a more negative experience of and attitude toward them, which may partly be explained by the lack of knowledge about the private standards and audits. Nevertheless, one main concern shared by official inspectors was the perception that private auditors too often ignored obvious animal welfare problems, and another concern mentioned was the risk of private auditors not being impartial due to financial interests. To what extent these perceptions are in accordance with reality, we cannot say based on this study. However, when several different private audit companies are competing for the same ‘customers’, there is a risk that the inspectors will be more lenient, which has, for example, become evident within the Swedish motor vehicle inspection market (54). Of the private standards within this study, KRAV has three different private audit companies carrying out animal welfare audits, from which the farmers can chose freely, and there may be a risk that the farmers chose the third-party audit company that has a reputation for being more lenient. It has also been stated that private standards are generally less reliable, and that there are challenges in relation to the control and enforcement of these (55). Now and then private standards have been investigated or scrutinized by animal rights organizations and media claiming that the audits are too lenient and do not keep what they promise in relation to their welfare standards or various marketing claims. For example, the RSPCA Assured certification is currently under pressure in the UK (56), and both Arlagården® and the organic KRAV audits have been questioned in Sweden (57, 58). However, it could also be argued that the presence of private standards and audits improves animal welfare and that it is an advantage to have inspections from different actors carrying out inspections independently from each other. Previous studies have, for example, shown that farmers who are affiliated with certain private standards and assurance schemes, hence being inspected by private auditors as well as official inspectors, have less non-compliance than other farmers during an official inspection (11, 59, 60).

The official inspectors recognized that animal keepers have difficulties in distinguishing between different inspections, i.e., mixing up private and official inspections. This was also recognized by the private auditors, but to a lower extent. Swedish dairy farmers have mentioned the risk of mixing different inspections up (15) and not understanding who is there for what inspection. Thus, the presence of these different inspections can make the work more difficult for the inspectors and auditors, as well as for the animal keepers, who are supposed to understand the role and aim of different inspections and different inspection outcomes.

Although the responding inspectors and auditors in this study had different views of the necessity of private audits in addition to official inspections, they agreed that collaboration and communication between them could and should be improved. It should be taken into account when discussing such matters, for example, that the official inspectors have a larger ‘toolbox’ to use when non-compliance is detected in order to enforce legislation (3). Furthermore, private audit companies can exclude members that do not comply with the basic requirements or membership rules of the private standard. Consequently, if an animal keeper is expelled from a private standard system, only the CABs will retain the task of enforcing the legislation with the former member and needs to be informed about this to be able to correct their risk classification of the farm in question. The dairy farmers and trotting horse trainers have already clearly stated that they would like to see better collaboration between the private audit companies and the CAB (15, 16).

It is worth noting that most of the respondents stated that it was reasonable to have an animal welfare inspection at least every third year at farms and other animal premises. This is in agreement with what the dairy farmers and trotting horse trainers stated (15, 16). However, the present goal, based on the resources available, for the official inspections is that 10% of farms with food producing animals are to be inspected each year (28), which means an inspection frequency of approximately every tenth year, although this may vary based on the nature of the risk-based system. Official control statistics show that the CABs do not even reach this goal (28). Even when they do reach this goal, it is still far from what the inspectors in this study thought would be reasonable. Private audits are carried out much more often, for example, KRAV carries out annual inspections at animal premises, which is more in accordance with what the respondents (inspectors, auditors and animal keepers) have stated as desirable. Public, governmental operations often struggle with cutbacks and limited resources, which means that priorities must be made (33). Animal welfare inspection activity is no exception. In addition to extended inspection intervals, this is also reflected in this study by the low grade of extra inspections (i.e., follow-up inspections due to non-compliances) that the official inspectors make compared with the private auditors, a phenomenon which has also been reported previously (11). In a French study, the authors found that compliance was improved at farms that were re-inspected but not at other farms (61). Hence, from this perspective, it could be argued that the presence of private standards contributes to ensure animal welfare at a farm level.

### 4.5 Respondents and study limitations

The questionnaire was made available to all official animal welfare inspectors and the majority of the private auditors in Sweden. The exact number of potential respondents is unknown, but we estimate that there were approximately 250 official inspectors and between 30 and 50 private auditors active in this field at the time of this study according to information we received from the CABs and the private audit companies. Hence, the two groups are inevitably uneven in size. There were more female than male respondents in this study. In general, women tend to reply to questionnaires more often than men (62). However, women are also considerably overrepresented among inspectors and auditors in Sweden. Response rates were relatively high, with a high proportion of completed answers, and the respondents covered all 21 counties of Sweden. A potential bias of respondents compared to the whole group of inspectors and auditors cannot be excluded (e.g., those more interested in the subject for various reasons), but unfortunately, we do not have access to descriptive statistics for the non-responders to further investigate this.

Statistical tests were performed on many questions, comparing responses between both inspectors and auditors, between female and male respondents, and between age groups of the respondents. We have strived to scrutinize and show the relevance of detected statistical differences by comparing with results from other scientific studies.

## 5 Conclusion

Swedish official inspectors and private auditors carrying out animal welfare inspections usually enjoy their work. They also seem to have quite similar ambitions and views on how to carry out inspections, aiming to create good dialog with the animal keeper. However, there are certain differences regarding how the inspections are perceived. For example, the official inspectors responded more frequently that they are exposed to unpleasant and threatening situations than the private auditors. They also have a slightly more negative view of the animal keepers’ knowledge about animal welfare, of how the animals are housed and managed, and of how the animal keepers act during the inspections. It should be borne in mind that the official inspectors also carry out inspections after complaints, often make unannounced inspections, inspect a greater range of animal premises (e.g., different pet premises alongside farm and horse premises), and have a position of greater power as representatives of the government than the private auditors. We suggest that these circumstances, together with differences in age and gender distribution, can potentially explain some of the differences in views between the official inspectors and the private auditors. The official inspectors also had a slightly more negative attitude toward the presence of private auditors than the other way around. Nevertheless, the respondents agreed that their communication and collaboration need to be improved.

This study can contribute to scientifically based discussions on working environment issues during the training of inspectors. It can also contribute to what further training should focus on, and how employers can manage the working environment in a more active way. Further research in this area is important as communication and information about inspections can be improved between authorities, private audit companies, and animal keepers so that expectations around animal welfare inspections are more aligned.

## Data Availability

The raw data supporting the conclusions of this article will be made available by the authors, without undue reservation.
