# Supplementary material for: Official and private animal welfare inspectors’ perception of their own on-site inspections
Source: Front Vet Sci. 2025 Apr 25;12:1575471. doi: 10.3389/fvets.2025.1575471 (PMC12062023; doi:10.3389/fvets.2025.1575471)
Supplement: Supplementary file 1 [file Data_Sheet_1.PDF]

Compiled version of questionnaire translated from Swedish to English

A questionnaire on expectations and experiences related to carrying out animal welfare inspections in Sweden

There is an ongoing research project at the Swedish University of Agricultural Sciences (SLU) on how animal keepers and inspectors perceive different kind of animal welfare inspections. This questionnaire is addressed to you, as an animal welfare inspector working with official inspections at a County Administrative Board (CAB), or to you as a private inspector auditing private regulations like Arlagården®, KRAV or the Trotting Health Standard.

The questionnaire is voluntary and no identifying information on you will be collected, i.e. we will not be able to see who has responded.

The aim with the project is to gain a greater understanding of your and your colleagues' experiences of carrying out animal welfare inspections, what insight you have into the inspection activities of other actors carrying out inspections at the same animal premises and how things could be done to improve the inspections.

The questionnaire consists of three parts:
- information on you and your professional role
- your expectations and experiences as an official inspector/private auditor
- your knowledge concerning other inspections/audits that covers animal welfare

Part 1. Information on you and your professional role

1. In which county in Sweden do you work?

2. If a private auditor: Which of the following regulations do you inspect concerning animal welfare?
   KRAV
   Arlagården®
   Trotting Health Standard
   Other____

3. If you make KRAV audits, for which certification body do you work?
   Kiwa
   SMAK
   HS Certifiering

4. How old are you?

5. Gender?

6. What is your highest level of education?

7. What education have you completed?

8. Have you attended any training/further training in the last five years?

9. Are you allowed (for your employer) to attend advanced and further training to the extent you consider necessary?

10. Is there advanced and further training to the extent you consider necessary?
    If not – what are you missing? *[optional free-text field]*

11. How much experience do you have of the following animal species/husbandry systems?

    Cattle
    Sheep/goats
    Pigs
    Poultry
    Other farm animal
    Horses
    Dogs
    Cats
    Other pet animal

12. How well do the following statements fit in with why you chose to become an inspector/auditor?

I want to help animals
I want to help humans

13. Which of the following statements about the quality of the inspection/audit business do you think best matches reality?
At our workplace, the focus is on the quality of the inspections/audits.
In our workplace, the focus is on quantity (i.e. the number of inspections/audits).
In our workplace, there is a good balance between quality and quantity.

14. Is your salary level affected by how many inspections/audits you do per year?

15. How would you like to describe your physical health?

16. How would you like to describe your mental health?

17. Do you enjoy being an inspector/auditor?

18. Do you have any plans to stop working as an inspector/auditor?

19. How many years have you been working as an inspector/auditor?

20. Does your boss have good knowledge of, and shows understanding of, the work you do?

21. In addition to animals being healthy and receiving good quality feed and water, what additional factors do you consider to be most important for animal welfare?
Tick the boxes of the 3 factors you think are most important.

That animals that are injured/fall ill quickly receive treatment
That animals have plenty of space
That animals are kept loose housed, i.e. not tied-up in stalls
That there is good air quality in the animal premises
That they get good care and management
That they feel safe with us who take care of them
That they are allowed to behave naturally
That they get daily access to outdoor runs/paddocks
That they have access to summer pasture
That they get physical activity / exercise
That they get the opportunity for positive experiences, to feel positive emotions
That the animal welfare legislation is complied with
Other, namely (free text)

22. How often do you inspect/audit the following animal species/types of animal husbandry?

Dairy cows
Beef cattle
Pigs
Sheep/goats
Poultry

Horses
Reindeers
Fish
Slaughter houses
Animal transports
Minks
Laboratory animals
Zoos
Dogs
Cats
Other pet animals

23. What do you think characterizes a good animal welfare inspector/auditor?
Tick the 3 most important characteristics for you.

The inspector/auditor is knowledgeable about the regulations.
The inspector/auditor is knowledgeable about cows and dairy farming.
The inspector/auditor is knowledgeable about animal welfare.
The inspector/auditor is nice.
The inspector/auditor is knowledgeable in the administrative procedures and processing of cases/matters.
The inspector/auditor acts professionally.
The inspector/auditor makes uniform assessments, i.e. so that it will be the same for everyone.
The inspector/auditor is confident in his/her assessments.
The inspector/auditor shows understanding that the animal keeper am under time and financial constraints.
The inspector/auditor shows understanding that minor deficiencies can always occur.
The inspector/auditor shows interest in the animals and the business.
The inspector/auditor is good at listening.
The inspector/auditor justifies and explain any non-compliances so that the animal keeper understand why it is important to take action.
The inspector/auditor complies with the regulations.
The inspector/auditor is smooth and can make flexible assessments as long as the animals are well.
The inspector/auditor gives advice on how the animal keeper can live up to the regulations.
The inspector/auditor gives advice about animal husbandry and activities that extend beyond the regulations.
Other, namely *[Free text]*

Part 2. Your expectations and experiences as an official/private inspector. If you carry out inspections based on several regulations you have to choose one of them.

24. If a private auditor: Which of the following regulations will you base your answers on?
KRAV
Arlagården®
Trotting health standard

25. Below are a number of statements regarding how you perceive the controllability of the regulation. Rate the statements on a scale from 1 (strongly disagree) to 5 (strongly agree):

It is easy to interpret the requirements.
I often feel uncertain in my assessments.
It is easy for me to make uniform assessments between different animal keepers.
My colleagues and I almost always make the same (uniform) assessments.
It is important that inspectors/auditors make uniform assessments.
It is easier to assess the animal-based requirements than the resource-based ones.
It is easier to assess the goal-oriented requirements than the resources-oriented.
It happens that I accept non-compliances if I assess that animal husbandry is generally good.

26. Are there requirements in the regulation that you find complicated or difficult to interpret?

27. Are there requirements in the regulation that you do not consider to benefit the welfare of animals in practice?

28. Do you feel that you have any opportunity to influence the development of the regulation?

29. How much do you agree or disagree with the following statements regarding the initiation of an inspection/audit? Rate the statements below on a scale from 1 (strongly disagree) to 5 (strongly agree).

Unannounced inspections/audits promote animal welfare more effectively than announced.
Announced inspections/audits contribute to a more pleasant atmosphere and better dialogue with animal keepers.
Today's system for the selection of who to inspect/audit promotes animal welfare in the best way.

30. How do you perceive the animal keepers that you carry out inspections/audits at? Consider a planned routine inspection/audit. Rate the following statements on a scale from 1 (strongly disagree) to 5 (strongly agree):

Most people have good animal husbandry.
Most people want their animals well.
Most people understand the purpose of the inspection/audit.
Most people seem calm and safe when I come and do an inspection/audit.
Most people listen to me.
Most people understand what I'm saying.
Most people act nice and professional.

Most people have good knowledge of the regulations.
Most people have good knowledge of animals' needs and animal welfare.
Most people want to be law-abiding and comply with the regulations.
Most animal husbandry is without any serious non-compliances.
Most animal husbandry is completely without non-compliances.
Most animal keepers agree with me in my assessments.
In most cases, discussions arise about how the regulations should be interpreted.
Most people understand what I write in the written inspection/audit report.
Most people ask for advice on how non-compliances can be remedied so that the regulations are complied with.
Most people ask for advice on things that go beyond the regulations.

31. Below are a number of statements regarding which tasks you consider to be included in your role as inspector/auditor. Rate the statements on a scale from 1 (strongly disagree) to 5 (strongly agree):

I am there to check compliance with the regulation.
I am there to help the animal keeper to live up to the requirements.
I am there to inform the animal keeper regarding new requirements.
I must give the animal keeper information so that he/she understands the meaning of the regulation.
I can give the animal keeper advice on the measures he/she needs to take to comply with the regulation.
I can give advice to the animal keeper so that the animal husbandry can be improved beyond the level of the regulation.

32. What do you feel are common reasons why an animal keeper does not comply with a regulation? Rate the statements below on a scale from 1 (strongly disagree) to 5 (strongly agree).

Lack of knowledge about the animals' needs is a common cause.
Lack of knowledge of the regulation is a common cause.
Lack of a will to comply with the regulation is a common reason.
Lack of ability to consider animals as sentient beings is a common cause.
Lack of practical and financial ability to comply with the regulations is a common reason.
Personal/psychological problems are a common cause.

[This question was repeated for "farmers", "horse keepers" and "pet keepers" in the questionnaire to official inspectors]

33. How much do you agree or disagree with the following statements regarding the handling of non-compliances during an inspection/audit? Rate the statements below on a scale from 1 (strongly disagree) to 5 (strongly agree).

It is important that I can justify why something is seen as a non-compliance.
It is important to be able to leave clear information during the inspection/audit about which non-compliances I have found.
It is acceptable to bring difficult assessment cases back to my colleagues as long as I give feedback to the animal keeper of the conclusion before sending out the inspection/audit report.

It is important to ensure that the animal keeper really understands what he/she must achieve.
It is acceptable to give a verbal statement for minor non-compliances without noting it in the checklist or in the inspection/audit report.
A non-compliance is a non-compliance regardless of severity so I handle them all equally.
Non-compliances involving several animals are more serious than if they concern a single animal.
It is important to let the animal keeper have a say when it comes to how long he/she should have to remedy the non-compliances.
We have predetermined time spans for when certain types of non-compliances must be rectified.
I believe that we often give animal keepers too many chances to correct non-compliances.
It is important to always follow up that the non-compliances have been corrected.
We always follow up that the non-compliances have been remedied.
We often trust that the animal keepers will correct the non-compliances and therefore we do not do a follow-up inspection/audit.

34. How much do you agree or disagree with the following statements regarding dialogue etc. during the inspections/audits? Rate the statements below on a scale from 1 (strongly disagree) to 5 (strongly agree).

Effective inspections/audits often leads to conflicts.
Conflicts with animal keepers are an obstacle to improve animal welfare.
Good dialogue is a prerequisite for a good inspection/audit.
It is important to me to create a safe and open atmosphere during an inspection/audit.
I often manage to create a relaxed atmosphere during the inspection/audit.
It often happens that animal keepers have shown me appreciation after an inspection/audit.
As an inspector, you are respected because of your competence regarding the regulation.
As an inspector, you are respected because of your competence regarding current animal husbandry and/or production methods.
As an inspector, you are respected because of your treatment of the people you inspect.
As an inspector, you are respected because of the sanction possibilities you have via the regulation.

35. Have you experienced difficult situations in connection with an inspection/audit? Rate the statements below on a scale from never to very often: (Never – Sometimes – Quite often – Often – Very often – Don't want to answer)

I have felt scared during an inspection/audit.
I have been subjected to threats in connection with my professional practice.
I have been exposed to physical violence in connection with my professional practice.
My competence has been questioned in connection with an inspection/audit.
I have received disparaging comments related to my age or gender.
I have seen animals subjected to great suffering.
I have seen people who have been in very difficult situations.

36. How much do you agree or disagree with the following statements regarding the occurrence and handling of troublesome situations in connection with an inspection/audit? Rate the statements below on a scale from 1 (strongly disagree) to 5 (strongly agree).

There is often a connection between poor animal husbandry and poor relationships with other people.
As an inspector/auditor, one must take into account an inspected person's personal problems and adapt assessments and decisions accordingly.
There are routines for how we should act when we see animal keepers who have severe personal problems.
There are routines that we should contact the Federation of Swedish Farmer's (LRF's) care group when a farmer needs support and help.
My employer has routines for dealing with threats and violence.
My employer has routines for dealing with difficult experiences during inspections/audits (e.g. seeing animals or people suffering or being questioned as an inspector/auditor).
The routines developed for difficult situations are followed.
There are routines that we must contact the county administrative board's animal welfare department when we see animal keepers who are unwell and this affects animal husbandry *[this statement was only given to the private auditors].*
Requesting assistance from the police works well in our county *[this statement was only given to the official inspectors].*

37. If you have been subjected to threats or violence at work, how well do the following statements agree? Rate the following statements on a scale from 1 (strongly disagree) to 5 (strongly agree):

The incident was prosecuted by my employer or me.
I feel that the incident affected my continued handling of the inspection case and the assessment and decisions I made.

38. Animal welfare is sometimes mentioned both in the media and in social media. Rate the statements below regarding what the media reports on a scale from 1 (strongly disagree) to 5 (strongly agree).

What the media reports about our inspections/audits usually matches reality well.
I myself have been an inspector/auditor in a matter that the media reported on.
I have been negatively mentioned by name on social media in connection with an inspection/audit.
I have been mentioned negatively, but without my name being given, on social media in connection with an inspection/audit.
I have been positively mentioned by name on social media in connection with an inspection/audit.
I have been positively mentioned, but without my name being given, on social media in connection with an inspection/audit.
I am not at all influenced by what is written in the media or on social media.

39. Regulations require that certain interpretations and assessments are made in individual cases, both in terms of determining whether something should be considered a non-compliance and in terms of how non-compliance should be handled. To what extent do you feel that the following factors influence your assessments and handling of an inspection/audit?
Rate the statements below on a scale from 1 (no influence at all) to 5 (very strong influence)

The overarching purpose and intent of the regulations.

What is stipulated in the specific requirements.
Written guidance (documents) from the rule owner
Guidance through email correspondence with the rule owner
Verbal guidance from the rule owner
Guidance developed jointly by multiple [certification bodies] / [CABs].
Guidance developed at my [certification body] / [CAB].
Discussions with my colleagues.
The animal keeper's attitude and ability to engage in dialogue.
The animal keeper's competence regarding animal protection and welfare.
The animal keeper's understanding and willingness to address deviations.
Experiences from previous inspections of this animal keeper.
The overall level of animal care (i.e., whether it is generally good or not).
My own condition on the day.
Signals from my immediate boss.
What is written in media/social media.

40. How much do you agree or disagree with the following statements about interpretive discretion? Rate the statements below on a scale from 1 (strongly disagree) to 5 (strongly agree).

    Inspectors/auditors should have significant interpretive discretion to find good solutions in individual cases.
    Inspectors/auditors should not have significant interpretive discretion because it leads to too much unequal treatment of different animal keepers.
    Inspectors/auditors should not have significant interpretive discretion because it reduces legal certainty.
    Inspectors/auditors should not have too much interpretive discretion because it could lead to excessively strict requirements on animal keepers.
    Inspectors/auditors should not have significant interpretive discretion because it could lead to weaker animal protection.

41. Below are a number of statements about satisfaction and usefulness regarding an inspection/audit. Rate on a scale from 1 (strongly disagree) to 5 (strongly agree):

    I am satisfied after an inspection/audit when I and the animal keeper have had a good dialogue.
    I am satisfied after an inspection/audit when I and the animal keeper are in agreement.
    I am satisfied after an inspection/audit when the animal keeper seems to have understood and absorbed what I said.
    I am generally satisfied after an inspection/audit when I have identified non-compliances that need to be addressed because I have then been useful.
    An inspection/audit carried out without finding any non-compliances feels unnecessary

42. Below are a number of statements about the tools and systems you use in your inspection/audit work. Rate on a scale from 1 (strongly disagree) to 5 (strongly agree):

    Our data system for inspections/audits works well.
    The checklists we use work well.
    I always fill in the checklist during my inspections/audits.
    I always fill in the checklist digitally during the inspection/audit.

We have the measuring equipment we need to perform our inspection/audit tasks.

43. Below are a number of statements about how you perceive the guidance provided to support your and your colleagues' assessments. Rate the statements on a scale from 1 (strongly disagree) to 5 (strongly agree)

    The inspection/audit guidance available from the rule owner is helpful when I make my assessments.
    My [certification body] / [CAB] has developed its own checklists to use during inspections/audits.
    My [certification body] / [CAB] has partially developed its own guidance/calibrations to ensure we can make consistent assessments.
    I have partially created my own checklists or guidance to feel confident in my own assessments.
    I have good support from my colleagues when I need help with my assessments.
    We conduct shadow inspections/audits of each other to improve consistency/calibration in our assessments.
    We receive good feedback regarding changes in assessment practices, i.e., when we need to change the way we make certain assessments

44. Do you have any suggestions for improving the assessment guidance?
    (This can include both written guidance and other forms of guidance methods, calibrations, training activities, etc.)
    No
    Yes, specify *[mandatory free-text field]*

45. On a scale from 1 (very negative) to 5 (very positive), how did you perceive the most recent inspection/audit you conducted?

Part 3. Your knowledge concerning other inspections/audits that covers animal welfare

46. Today, many animal keepers are subject to inspections/audits both by the County Administrative Board and by private actors such as KRAV, Arlagården®, IP Sigill, and the Trotting Health Standard.
    Below are a number of general statements regarding the existence of various regulations. Please rate them on a scale from 1 (strongly disagree) to 5 (strongly agree).

    It is necessary to have both official and private inspections/audits regarding animal welfare.
    The presence of private standards is good for animal welfare.
    It is easy for animal keepers to differentiate between the various official and private inspections/audits conducted on their operations.
    I believe it is good that participation in a private regulatory framework is included as a factor in the County Administrative Board's risk assessment, i.e., that those inspected by private actors may be inspected less frequently by the County Administrative Board.
    I am well-informed about the regulations (i.e. both private and official) concerning animal welfare that exist.
    I am well-informed about the inspections/audits, beyond those related to animal welfare, that the animal keepers I inspect are also subject to.

    If you would like to clarify anything regarding your views on the existence of private standards, write it here. *[optional free-text field]*

47. Below are a number of general statements regarding the content and assessments of various regulations. Please rate them on a scale from 1 (strongly disagree) to 5 (strongly agree).

    I am well-informed about the similarities and differences between the legislation and the private standards regarding requirements and assessments.
    I usually inform the animal keeper during an inspection/audit that other inspecting bodies may make different assessments than I have.
    I believe that private auditors make stricter animal protection assessments than the County Administrative Board.
    I believe that private auditors and the County Administrative Board often make the same assessments regarding similar requirements, i.e., often reach the same conclusions.
    I am aware of what decisions on actions and sanctions other actors can make if they identify non-compliances during their inspections/audits.

    If you would like to clarify anything regarding the content and assessments of different regulations, write it here. This could include, for example, that you believe a particular regulation stands out compared to others or that you have more knowledge about specific regulations.
    [optional free-text field]

48. Below are a number of general statements regarding the control frequency and synchronization of private audits and official inspections. Please rate them on a scale from 1 (strongly disagree) to 5 (strongly agree).

I am well-informed about how often private actors conduct their audits, i.e., their audit frequency.
I am well-informed about how often the County Administrative Board conducts its inspections, i.e., its inspection frequency.
At my workplace, we try to synchronize our inspections/audits with those of the County Administrative Board/private actor for the same animal keeper, so that our visits are spread out over time.
At our workplace, we aim to conduct inspections/audits simultaneously with the County Administrative Board/private actor to reduce the number of inspection visits for an animal keeper.
At our workplace, we have a routine to contact the County Administrative Board/private actor if we note animal protection issues during an inspection.
The County Administrative Board/private actor often informs us if they identify animal welfare non-compliances during their inspections/audits.
I would like to see better cooperation among those conducting private audits *[this statement was only given to the private auditors]*.
I would like to see better cooperation between the County Administrative Board and private actors.
During an audit, I have received the comment from the animal keeper that “you were just here,” but it turned out it was another private actor that had conducted an audit *[this statement was only given to the private auditors]*.
During an inspection, I have received the comment from the animal keeper that “you were just here,” but it turned out it was another official inspector (from another field) that had conducted an inspection *[this statement was only given to the official inspectors]*.
During an audit, I have received the comment from the animal keeper that “you were just here,” but it turned out it was an official inspection (County Administrative Board) that had been conducted *[this statement was only given to the private auditors]*.
During an inspection, I have received the comment from the animal keeper that “you were just here,” but it turned out it was a private auditor that had been conducted an audit *[this statement was only given to the official inspectors]*.

If you would like to clarify anything regarding the control frequencies and synchronization between different actors, write it here. This could include, for example, that you/your workplace has closer cooperation with certain control actors than others.
[optional free-text field]

49. How often do you think it is generally reasonable for an animal keeper to receive inspection/audit visits concerning animal welfare (either from the County Administrative Board or private actors)?
At least once a year
Once per year
Every two years
Every three years
Every four years
Every five years
Less frequently than every five years

50. Do you think someone should take clearer overall responsibility for the entire inspection system, including both private and official inspections/audits?
Yes

No
Don't know

If you answered yes, who do you see as having the main responsibility? *[optional free-text field]*

51. Finally – is there anything else you would like to share regarding inspections/audits of animal husbandry and regulations that we have not asked about? *[optional free-text field]*

Thank you very much for your participation!
